# Day-of-the-week variation in ischemic stroke admissions in patients with atrial fibrillation

**DOI:** 10.1080/07853890.2026.2667620

**Published:** 2026-05-11

**Authors:** Kasperi Nuopponen, Ville Langén, K. E. Juhani Airaksinen, Jussi Jaakkola, Eero Jalli, Olli Halminen, Jari Haukka, Jukka Putaala, Miika Linna, Pirjo Mustonen, Juha Hartikainen, Mika Lehto, Konsta Teppo

**Affiliations:** a Division of Medicine, University of Turku, Turku, Finland; b Division of Medicine, Turku University Hospital and University of Turku, Turku, Finland; c Turku University Hospital and University of Turku, Turku, Finland; d Department of Internal Medicine, Satasairaala, Pori, Finland; e University of Eastern Finland, Kuopio, Finland; f University of Helsinki, Helsinki, Finland; g Neurology, Helsinki University Hospital and University of Helsinki, Helsinki, Finland; h Aalto University, Espoo, Finland; i Kuopio University Hospital and University of Eastern Finland, Kuopio, Finland; j Jorvi Hospital, Department of Internal Medicine, HUS Helsinki University Hospital and University of Helsinki, Helsinki, Finland

**Keywords:** Atrial fibrillation, circaseptan variation, stroke, weekday variation

## Abstract

**Background:**

No data exist on whether ischemic stroke admissions among patients with atrial fibrillation (AF) vary by day of the week.

**Methods:**

The nationwide registry-linkage FinACAF study includes all patients with incident AF in Finland between 2007 and 2018. This analysis focused on patients who experienced their first-ever ischemic stroke. Hospital stroke admissions were categorized by day of the week, and a weekday-to-weekend ratio was calculated as the ratio of strokes on a weekday versus a weekend day.

**Results:**

We identified 13 781 patients (mean age 79.2 years; 57.1% women) with AF admitted for ischemic stroke. Stroke admissions varied significantly by day of the week (*p* < 0.001), with the highest number occurring on Mondays (16.1% of all strokes), followed by a progressive decline over the week. Admission rates were notably higher on Monday and Tuesday, remained relatively stable from Wednesday to Friday, and decreased on Saturday and Sunday (11–12% of all strokes per day). The overall weekday-to-weekend ratio was 1.31 (95% CI 1.26–1.37) and was more pronounced in men than in women, as well as during the first half of the study period (2007–2012) compared to the latter half (2013–2018).

**Conclusions:**

This nationwide cohort study demonstrates a clear day-of-the-week variation in hospital admissions for ischemic stroke among patients with AF, with fewer admissions on weekends than on weekdays. Promoting awareness of stroke symptoms and the importance of seeking timely care regardless of the day may represent a modifiable target to improve outcomes in patients with AF who experience stroke.

## Graphical Abstract

**Figure:**
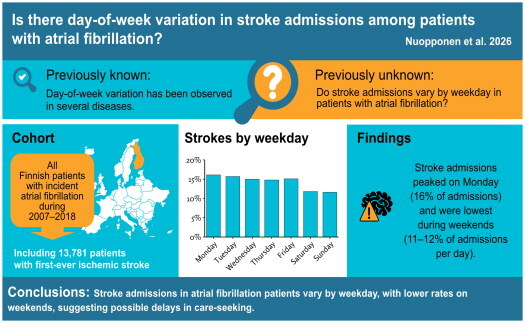

## Introduction

Atrial fibrillation (AF) is the most common sustained cardiac arrhythmia, affecting up to 5.2% of the adult population [1–3]. It is a major cause of ischemic stroke, a condition that ranks as the second leading cause of death worldwide [4,5]. Treatment of acute ischemic stroke has advanced substantially over recent decades, and prompt medical evaluation of stroke symptoms is essential to enable time-critical acute therapies, such as thrombolysis and thrombectomy, in eligible patients. Early stroke unit treatment is also vital in improving patient outcomes [3].

Previous studies have reported day-of-the-week variations in the incidence of various conditions, such as sudden unexpected cardiac death and ischemic stroke [6–10]. Potential factors influencing day-of-the-week variation may include circadian rhythm disturbances, work-related stress, or other biological mechanisms, as well as factors influencing patients’ care-seeking behaviour on weekdays versus weekends, and differences in the accessibility of medical services [11–14].

However, regarding ischemic stroke specifically in patients with AF, research on day-of-the-week variation is lacking. The pathophysiology in AF-related ischemic strokes (i.e. the dislocation of an embolus primarily from the left atrial appendage) differs from other main stroke subtypes, and so AF-related strokes could have a unique circaseptan pattern. Furthermore, we hypothesised that differences in care-seeking behaviour or reduced healthcare accessibility during weekends may delay stroke diagnosis and contribute to day-of-the-week variation in stroke admissions. Therefore, we conducted this retrospective nationwide cohort study to investigate whether day-of-the-week variation exists in ischemic stroke admissions in patients with AF.

## Methods

### Study population

The Finnish AntiCoagulation in Atrial Fibrillation (FinACAF) Study (ClinicalTrials Identifier: NCT04645537; ENCePP Identifier: EUPAS29845) is a nationwide retrospective cohort study that includes all patients documented with AF in Finland from 2004 to 2018 [15]. Patients were identified using all available national healthcare registers, including hospitalizations and outpatient specialist visits (Hilmo), primary healthcare (Avohilmo), and the National Reimbursement Register maintained by the Social Insurance Institute (KELA). The cohort inclusion criterion was the International Classification of Diseases, 10th Revision (ICD-10) diagnosis code I48, encompassing atrial fibrillation and atrial flutter, collectively referred to as AF, recorded between 2004 and 2018. Exclusion criteria included permanent emigration abroad before December 31, 2018, and an age of under 20 years at the time of AF diagnosis. The present sub-study was conducted within a cohort of patients with incident AF from 2007 to 2018, originally established in earlier studies of the FinACAF cohort and adapted for the purposes of the current study by excluding all patients who did not experience an ischemic stroke after the diagnosis of AF during 2007 and 2018 [16–18]. The current study focused on the first-ever ischemic stroke, since defining the timing of recurrent strokes in registry data may be less reliable. Therefore, patients with a history of ischemic stroke before the onset of AF were excluded (Supplementary Figure 1). Definitions for baseline comorbidities are listed in Supplementary Table 1.

### Definition of ischemic stroke

An ischemic stroke event was defined as the first occurrence of an I63 or I64 ICD-10 diagnosis code in the hospital care register after AF diagnosis entry. Ischemic stroke diagnoses recorded only in the hospital register were included to ensure that the events captured were major and clinically relevant. Ischemic stroke events were categorized according to the day of the week of hospital admission.

### Study ethics

The study protocol was approved by the Ethics Committee of the Medical Faculty of Helsinki University, Helsinki, Finland (nr. 15/2017 and 15/2024) and received research permission from the Helsinki University Hospital (HUS/46/2018 and HUS/217/2024). Respective permissions were obtained from the Finnish register holders (KELA 138/522/2018, THL 2101/5.05.00/2018, Population Register Centre VRK/1291/2019-3, Statistics Finland TK-53-1713-18/u1281, and Tax Register VH/874/07.01.03/2019). Patients’ personal identification numbers were pseudonymized, and the research group received individualized but unidentifiable data. Informed consent was waived due to the retrospective registry nature of the study. The study conforms to the Declaration of Helsinki as revised in 2024.

### Statistical analyses

Differences in the prevalence of comorbidities were assessed using the chi-square test, while continuous variables were compared using one-way analysis of variance. The number and proportion of ischemic strokes occurring on each day of the week were calculated, and the ratio of strokes on a weekday versus a weekend day was determined. Differences in admission frequencies across weekdays were evaluated using a chi-square goodness-of-fit test assuming equal expected frequencies. Subgroup analyses were performed according to sex, age (<65 or ≥65 years), income tertiles, stroke year (2007–2012 or 2013–2018), prior use of oral anticoagulants, and rural versus urban residence. Binary logistic regression was then used to assess the association of factors with ischemic stroke admissions on weekdays (Monday to Friday) compared with weekends (Saturday and Sunday). Consequently, odds ratios above 1 indicate that the variable of interest is associated with a higher likelihood of stroke admission on a weekday. All statistical analyses were conducted using IBM SPSS Statistics version 28.0 (SPSS, Inc., Chicago, Illinois, USA) or R version 4.0.5 (R Core Team, Vienna, Austria; https://www.R-project.org).

## Results

We identified 13 781 patients (mean age 79.2 years; women 57.1%) with AF admitted with ischemic stroke. No clinically meaningful differences were observed in patient demographics or the prevalence of comorbidities according to the day of the week of stroke admission, although differences in some variables, such as sex, reached statistical significance (Table 1). Differences in patient characteristics were also similar when stroke patients were categorized by weekday versus weekend admission (Supplementary Table 2).

**Table 1.** Baseline characteristics of the study cohort stratified by day of stroke admission.

	Monday	Tuesday	Wednesday	Thursday	Friday	Saturday	Sunday	
	*n* = 2221	*n* = 2161	*n* = 2063	*n* = 2040	*n* = 2078	*n* = 1622	*n* = 1596	*p* value
Mean age, years	79.0	79.4	79.2	78.7	78.1	79.5	79.5	0.173
Female sex, %	56.8	58.5	56.6	55.0	55.9	60.0	58.7	0.028
**Income tertiles**								0.536
1^st^ (lowest), %	35.6	33.2	31.5	33.1	33.1	33.2	34.1	
2^nd^, %	32.5	33.4	34.7	33.4	32.9	32.5	33.8	
3^rd^ (highest), %	32.0	33.4	33.8	33.5	34.0	32.0	32.1	
**Comorbidities**								
Any vascular disease	32.1	30.8	29.5	29.4	30.8	29.4	28.7	0.267
Prior MI	9.9	10.2	8.6	8.7	8.8	9.8	8.8	0.334
Diabetes	17.4	16.2	14.4	15.1	14.4	11.6	11.0	0.259
Dyslipidemia	16.2	15.3	15.2	14.8	15.2	11.9	11.5	0.898
Hypertension	77.5	79.8	75.4	76.4	76.7	78.0	78.4	0.021
Congestive heart failure	20.7	19.1	18.5	18.2	18.7	19.9	18.0	0.298
Abnormal liver function	0.5	0.4	0.2	0.3	0.5	0.4	0.3	0.823
Abnormal renal function	4.0	4.1	2.9	3.5	3.0	3.2	3.7	0.215
Alcohol use disorder	3.9	4.1	4.5	4.6	4.4	3.6	4.4	0.771
Dementia	4.9	5.6	4.2	3.8	4.6	6.0	4.7	0.030
Prior bleeding	11.2	10.7	11.5	11.3	11.5	9.8	12.3	0.409
Antiplatelets or NSAIDS	30.6	28.8	27.9	31.7	29.0	29.0	29.5	0.133
Coronary heart disease	25.0	25.2	24.1	23.2	23.6	24.6	22.7	0.463
Cancer	20.6	21.5	21.2	20.0	20.7	20.5	20.1	0.905
Psychiatric disease	11.7	11.0	11.8	11.7	11.5	11.0	12.4	0.882
OAC	55.8	54.5	56.8	55.5	55.0	56.8	55.3	0.745
**Risk scores**								
Mean modified HAS-BLED score	2.5	2.5	2.4	2.4	2.4	2.5	2.5	0.329
Mean CHA_2_DS_2_-VASc score	3.7	3.7	3.6	3.5	3.6	3.7	3.6	0.005

Values denote proportions (%) or means (standard deviation).

MI, myocardial infarction; CHA_2_DS_2_-VA(Sc) score, congestive heart failure (1 point), hypertension (1 point), age ≥75 years (2 points), diabetes (1 point), history of stroke or TIA (2 points), vascular disease (1 point), age 65–74 years (1 point)(, sex category (female) (1 point)); modified HAS-BLED score, hypertension (1 point), abnormal renal or liver function (1 point each), prior stroke (1 point), bleeding history (1 point), age >65 years (1 point), alcohol abuse (1 point), concomitant antiplatelet/NSAIDs (1 point) (no labile INR, max score 8).

**Figure 1.**
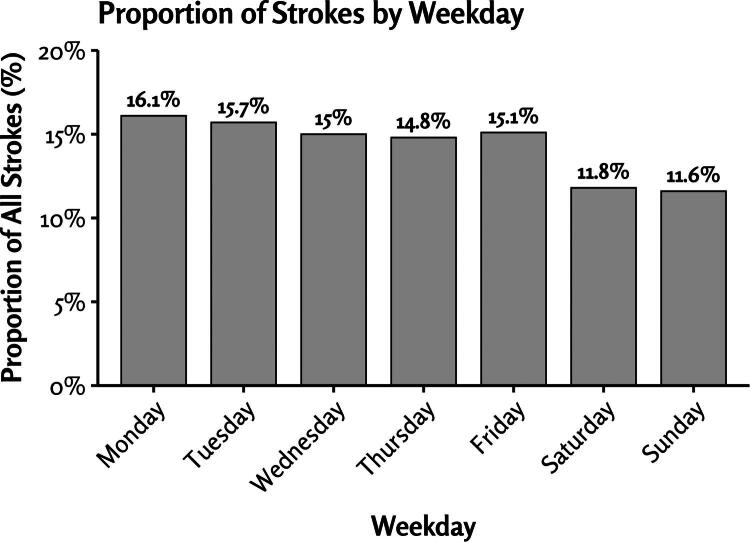
Proportion of strokes by weekdays.

**Figure 2.**
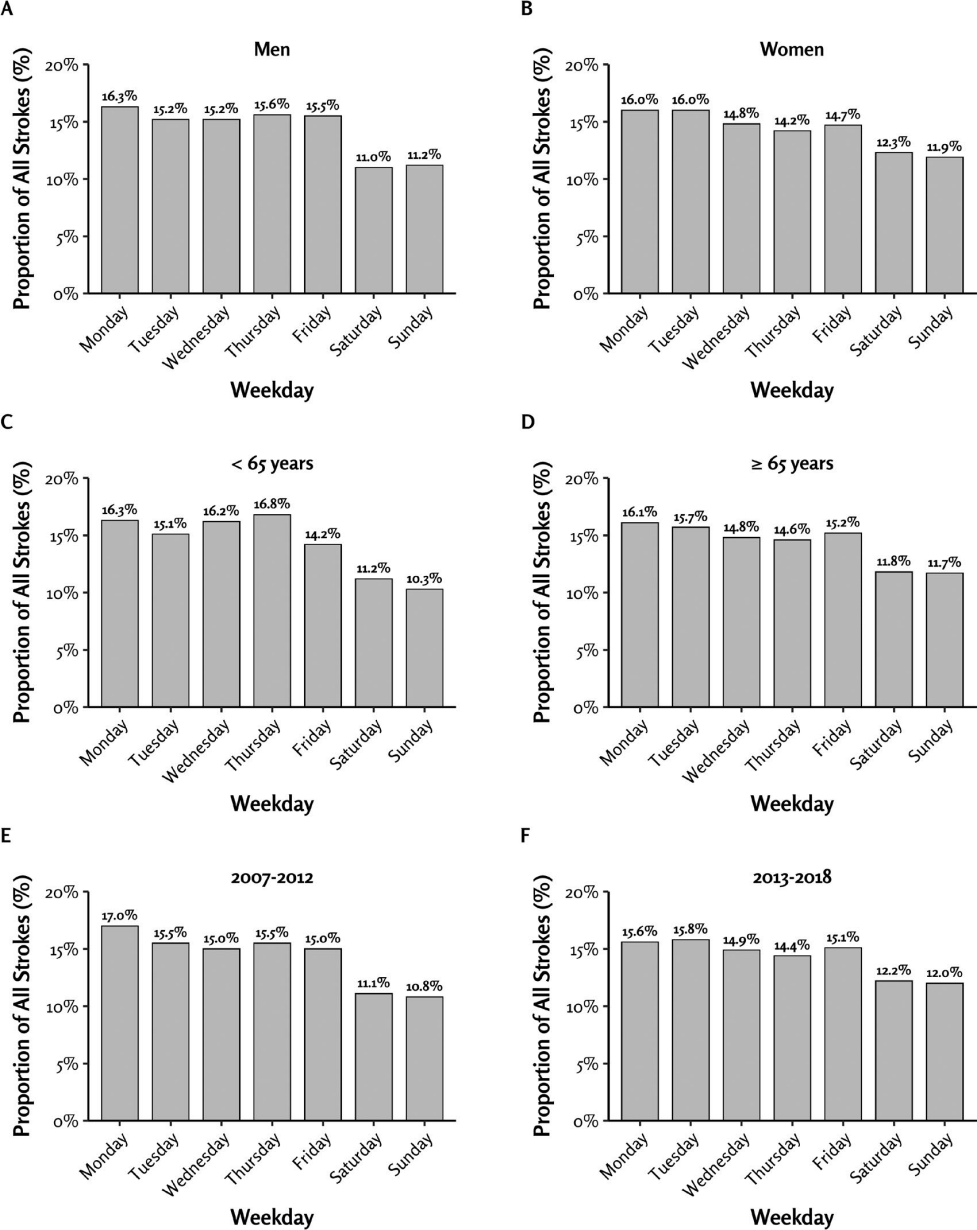
Proportion of strokes by weekdays by subgroups.

Ischemic stroke admissions varied significantly by day of the week (*p* < 0.001), with the highest number of admissions occurring on Mondays (16.1% of all strokes), which then declined progressively over the course of the week (Figure 1). Admission rates were notably higher on Monday and Tuesday, remained relatively stable from Wednesday to Friday, and showed a notable decrease on Saturday and Sunday (11–12% of all strokes per day). The majority of stroke admissions occurred during weekdays (76.6% of strokes; Figure 1). The average number of ischemic stroke admissions per weekday was 31% higher than the average number per weekend day (*p* value <0.001; Table 2). This pattern was observed among all studied subgroups (Figure 2; Supplementary Figure 2).

**Table 2.** Weekday-to-weekend ratios of stroke admissions.

Group	Ratio	95% CIs	*p* value
All	1.31	1.26–1.37	
**Gender**			0.003
Men	1.40	1.32–1.49	
Women	1.25	1.19–1.32	
**Age, years**			0.128
<65	1.46	1.28–1.66	
65+	1.30	1.25–1.35	
**Income tertiles**			0.313
Income 1	1.31	1.21–1.39	
Income 2	1.27	1.21–1.37	
Income 3	1.39	1.27–1.46	
**Year of stroke**			0.001
2007–2012	1.43	1.33–1.53	
2013–2018	1.25	1.20–1.32	
**OAC status**			0.152
OAC Before stroke	1.31	1.23–1.37	
No OAC before stroke	1.38	1.25–1.41	
**Residence**			0.379
Rural	1.29	1.21–1.38	
Urban	1.33	1.27–1.40	

CI, confidence interval; OAC, oral anticoagulant. *p* values refer to the statistical differences in the ratios between subgroups. Ratios are calculated as stroke admissions per weekday divided by stroke admissions per weekend day.

The weekday–weekend ratio was slightly more pronounced among men (*p* value 0.003 compared with women) and among patients younger than 65 years, although the difference between those younger and those older than 65 did not reach statistical significance (*p* value 0.128; Table 2; Figure 2). Moreover, before 2013, ischemic stroke admissions occurred 43% more often on weekdays compared with weekend days. This difference attenuated over time, and from 2013 onwards, strokes occurred 25% more frequently per weekday (Table 2; Figure 2).

The weekday-to-weekend stroke ratio did not differ significantly by income, rural/urban residency, or prior OAC use (Table 2). However, a slightly higher peak in the proportion of stroke admissions on Mondays was observed among patients residing in rural areas and those in the lowest income tertile, compared with patients living in urban areas and those in the highest income tertile (Supplementary Figure 2).

When analyzing factors associated with stroke admission on a weekday, only a later year of stroke admission was significantly associated with a lower likelihood of weekday admission. Female sex showed borderline significance for a lower likelihood of weekday admission, while all other variables were not statistically significant in the multivariable analysis (Supplementary Table 3).

**Figure 3.**
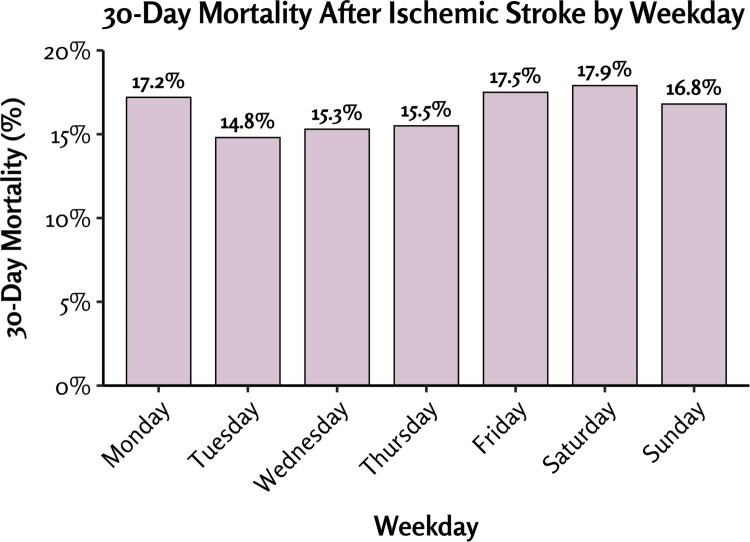
30-day mortality after ischemic stroke by weekday.

Overall, 2,258 patients (16.3%) died within 30 days after stroke. Mortality was highest among patients admitted on Mondays, Fridays, or Saturdays and lowest for those admitted from Tuesday to Thursday (*p* = 0.048; Figure 3).

## Discussion

This nationwide cohort study examined day-of-the-week variation in ischemic stroke admissions among patients with AF, demonstrating a significantly lower number of admissions on weekends. Although the weekday–weekend difference attenuated over the study period, during the most recent years, there were still, on average, 25% more ischemic stroke admissions per weekday compared with an average weekend day.

To our knowledge, no prior studies have specifically examined day-of-the-week variation in ischemic stroke admissions among patients with AF. However, other studies have reported day-of-the-week variations in sudden death, myocardial infarction, and ischemic stroke, with incidence peaking on Saturdays, while contradictory findings have also been reported, indicating a peak on Mondays [6,8,19]. Hospital admissions in general are known to decline during weekends, and our observation of reduced stroke admission rates during weekends is consistent with this trend [20,21]. Although the weekday–weekend ratio did not differ significantly by income or residence, patients with rural residence and low income exhibited a modest Monday peak, consistent with prior observations of higher stroke admission rates on Mondays among individuals of lower socioeconomic status in the general population [9].

Ischemic stroke is an acute condition that requires urgent treatment. Delaying medical attention until the next day or until Monday can result in major long-term consequences, including disability, cognitive decline, emotional changes, and physical impairments. The most important acute treatment options for ischemic stroke include thrombectomy and thrombolytic therapy, both of which are time-dependent and guided by the interval from symptom onset. After 9–24 h from stroke onset, the treatment window closes depending on the type of occlusion and salvageable tissue [22]. In addition, rapid recognition of stroke symptoms allows timely admission to stroke centers and specialized stroke units, which significantly improves outcomes and mortality among patients with stroke [23].

Factors underlying day-of-the-week variation are likely multifaceted. Although biological factors involved in the etiology of stroke, such as weekly variations in lifestyle, stress levels, and blood pressure, may in theory contribute to this phenomenon, they are unlikely to fully account for the observed magnitude of day-of-the-week variation in stroke admissions [6,8,24]. Moreover, the observed weekday-to-weekend admission pattern was present in both patients with and without prior oral anticoagulant use, suggesting that differences in anticoagulation status do not explain this pattern. Thus, it is probable that other factors, such as care-seeking behaviour or reduced health-care accessibility during weekends, play a role in the observed lower rate of stroke admissions during weekends [12,13]. For example, patients may have a higher threshold for seeking care for mild symptoms suggestive of stroke. Moreover, outdated public perceptions from the pre-thrombolysis and thrombectomy era may lead some, particularly older individuals, to underestimate the urgency of acute stroke care. The early-week peak in stroke admissions may reflect a compensatory excess from the lower-than-average weekend admissions and may also indicate that some patients inappropriately delay seeking care until Monday. Encouragingly, the disparity in stroke admissions between weekdays and weekends diminished over the study period. This trend may be attributable to public health initiatives promoting early recognition and response to stroke symptoms, heightened awareness of AF and AF-related stroke, and broader improvements in healthcare delivery and access. Despite these improvements, weekday–weekend difference in stroke admissions persisted even in the later phase of the study period. The results indicate that promoting awareness of stroke symptoms is important in order to encourage timely care-seeking irrespective of the day of the week [25]. This can represent an important modifiable target for improving outcomes in patients with AF who experience ischemic stroke.

Mortality after stroke was higher among patients admitted on Monday compared to those admitted between Tuesday and Thursday, and increased again for admissions on Friday and Saturday. The slightly higher mortality for Monday admissions may in part reflect delays in seeking care, but several other factors are likely involved, and causal interpretations should be made with particular caution. For example, the higher mortality during weekends could reflect that patients with more severe strokes presented to the emergency department more promptly because their symptoms were severe and could not be delayed. Differences in acute treatment between weekdays and weekends may also contribute, as suggested by previous studies [26–28].

## Strengths and limitations

A major strength of our study is the large sample size collected from all levels of healthcare and detailed patient information, particularly regarding cardiovascular diseases. In addition, the hospital care register used to identify stroke events is well-validated and has demonstrated high diagnostic accuracy, particularly for cardiovascular disease [29]. The main limitation of our study is that the administrative data lacked information on the onset of stroke symptoms, as well as the exact time of arrival at the emergency department or hospital admission. Furthermore, potential inaccuracies in the administrative data may introduce information bias. This study was conducted solely in Finland, which may limit the generalizability of the findings to other healthcare settings.

## Conclusion

In conclusion, this nationwide cohort study demonstrated a day-of-the-week variation in hospital admissions for ischemic stroke among patients with AF, with admissions occurring less frequently on weekends than on weekdays. Increasing awareness of stroke symptoms and encouraging patients to seek immediate medical attention, irrespective of the day of the week, may enable more timely treatment and improve outcomes in acute stroke.

## Supplementary Material

Supplementary_Material.docx

## Data Availability

Due to the sensitive nature of the data and the agreements made with Finnish register holders, the dataset collected for this study cannot be shared.
