# Supplementary material for: Day-of-the-week variation in ischemic stroke admissions in patients with atrial fibrillation
Source: Ann Med. 2026 May 11;58(1):2667620. doi: 10.1080/07853890.2026.2667620 (PMC13162540; doi:10.1080/07853890.2026.2667620)

**Supplementary Material**

**Supplementary Table 1.** Definitions of the comorbidities

**Supplementary table 2.** Cohort characteristics according to date of stroke (weekday vs. weekend).

**Supplementary table 3.** Factors associated with stroke admissions during weekdays (Monday to Friday) weekends (Saturday or Sunday)

**Supplementary Figure 1.** Flow chart of the patients selection process

**Supplementary Figure 2.** Proportions of strokes by weekday across subgroups defined by income, OAC use, and residence

|  | ICD-10 | ICPC-2 | Reimbursement code | ATC code | Other |
| --- | --- | --- | --- | --- | --- |
| Any vascular disease | I20-I25, I65-I66, I67.2, I70 | K74, K75, K76, K91, K92 | 206 |  |  |
| Hypertension | I10-I15 | K85, K86, K87 | 205 | C03A, C03B, C03DB, C03EA, C07A, C08CA, C08D, C09 |  |
| Dyslipidemia | E78 | T93 | 206 | C10 |  |
| Heart failure | I50, I11.0, I13.0, I13.2 | K77 | 201 |  |  |
| Diabetes | E10-E14 | T89, T90 | 103, 215 | A10 |  |
| Previous stroke | I63, I64, I69.3-I69.8 | K90 |  |  |  |
| Bleeding history | D50.0, D62, D68.3, I60-I62, I69.0-I69.2, I85.0, I86.4, J94.2, K22.1, K22.3, K22.6, K25.0, K25.2, K25.4, K25.6, K26.0, K26.2, K26.4, K26.6, K27.0, K27.2, K27.4, K27.6, K28.0, K28.2, K28.4, K28.6, K29.0, K62.5, K63.1, K63.3, K92.0-K92.2, N02, R04, R31, R58, S06.2-S06.6, S06.8 |  |  |  |  |
| Alcohol abuse | F10 |  |  |  |  |
| Renal failure or dialysis | N18, Z49 |  |  |  |  |
| Liver cirrhosis or failure | K70.2-K70.4, K71.7, K71.8, K72, K74 |  |  |  |  |
| Dementia | F00-F03, G30 |  |  |  |  |
| Cancer |  |  |  |  | Any cancer registered in the Finnish Cancer Registry |
| Psychiatric disorder | F04-F99 |  |  |  |  |

**Supplementary Table 1.** Definitions of the comorbidities

**Supplementary table 2.** Cohort characteristics according to date of stroke (weekday vs. weekend).

|  | Weekday (Monday to Friday) | Weekend (Saturday & Sunday) | p-value |
| --- | --- | --- | --- |
|  | n=10563 (77 %) | n=3218 |  |
| Mean age, years | 79.1 | 79.5 | 0.004 |
| **Sex** |  |  | 0.005 |
| Women | 56.6 | 59.4 |  |
| Men | 43.4 | 40.6 |  |
| **Comorbidities** |  |  |  |
| Congestive heart failure | 19.0 | 18.9 | 0.886 |
| Dementia | 4.6 | 5.3 | 0.096 |
| Psychiatric disease | 11.5 | 11.7 | 0.763 |
| OAC before stroke | 55.5 | 56.0 | 0.614 |
| Cancer before or at cohort | 20.8 | 20.3 | 0.527 |
| Coronary heart disease | 24.3 | 23.6 | 0.482 |
| Myocardial infarction | 9.3 | 9.3 | 0.913 |
| Any vascular disease | 30.6 | 29.1 | 0.106 |
| Antiplatelets or NSAIDS | 29.6 | 29.2 | 0.701 |
| Alcohol use disorder | 4.3 | 4.0 | 0.570 |
| Prior bleeding | 11.2 | 11.1 | 0.795 |
| Abnormal liver function | 0.4 | 0.4 | 0.903 |
| Abnormal renal function | 3.5 | 3.4 | 0.885 |
| Diabetes | 22.6 | 21.7 | 0.245 |
| Dyslipidemia | 46.0 | 45.9 | 0.956 |
| Hypertension | 77.2 | 78.2 | 0.235 |
| Mean CHA_2_DS_2_-VASc | 3.6 | 3.7 | 0.109 |
| Mean modified HAS-BLED | 2.5 | 2.5 | 0.408 |
|  |  |  |  |

**Supplementary table 3.** Factors associated with stroke admissions during weekdays (Monday to Friday) weekends (Saturday or Sunday)

| Variable | Unadjusted OR | Adjusted OR |
| --- | --- | --- |
| Age (per 10 year increase) | 0.96 (0.93–1.00) | 0.98 (0.94–1.02) |
| Female sex | 0.89 (0.82–0.97) | 0.92 (0.84–1.00) |
| Income tertile 2 | 0.99 (0.90–1.09) | 0.99 (0.90–1.09) |
| Income tertile 3 | 1.05 (0.95–1.15) | 1.03 (0.93–1.15) |
| Year of stroke admission (per 1 year) | 0.98 (0.97–0.99) | 0.98 (0.96–0.99) |
| Hypertension | 0.94 (0.86–1.04) | 0.96 (0.87–1.06) |
| Congestive heart failure | 1.01 (0.91–1.11) | 1.00 (0.90–1.11) |
| Hyperlipidemia | 1.00 (0.93–1.08) | 0.99 (0.90–1.08) |
| Diabetes | 1.06 (0.96–1.16) | 1.06 (0.96–1.17) |
| Abnormal renal function | 1.02 (0.82–1.27) | 1.00 (0.81–1.25) |
| Abnormal liver function | 1.04 (0.56–2.07) | 1.01 (0.54–2.01) |
| Bleedings | 1.02 (0.90–1.15) | 1.01 (0.89–1.15) |
| Alcohol use disorder | 1.06 (0.87–1.30) | 1.06 (0.83–1.36) |
| Any vascular disease | 1.07 (0.99–1.17) | 1.07 (0.98–1.18) |
| Cancer | 1.03 (0.94–1.14) | 1.04 (0.95–1.15) |
| Dementia | 0.86 (0.72–1.03) | 0.88 (0.73–1.06) |
| Psychiatric disease | 0.98 (0.87–1.11) | 0.97 (0.84–1.13) |
| OAC before stroke | 0.98 (0.90–1.06) | 1.01 (0.93–1.09) |
|  |  |  |

**Supplementary Figure 1.** Flow chart of the patients selection process


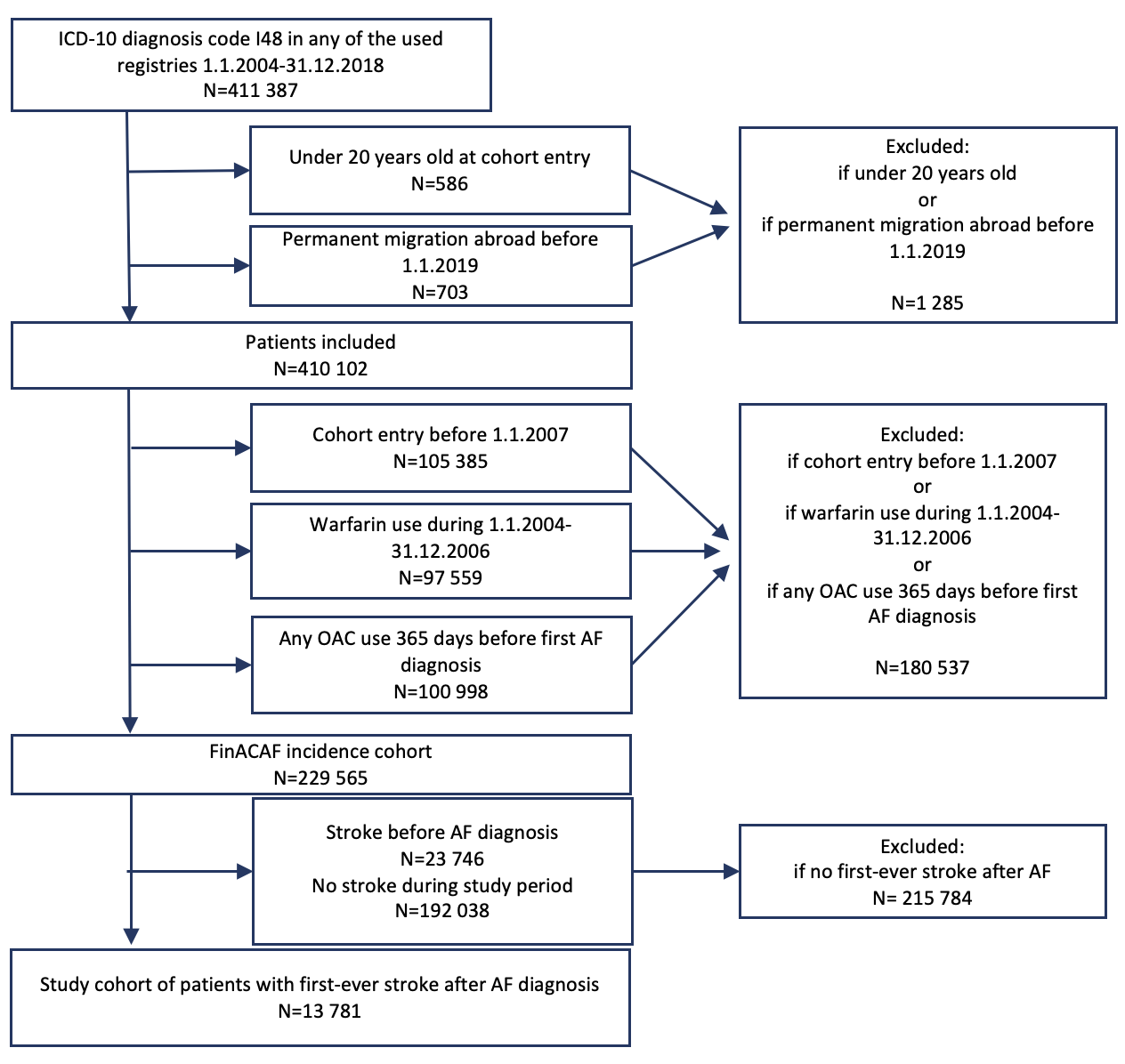


**Supplementary Figure 2.** Proportions of strokes by weekday across subgroups defined by income, OAC use, and residence


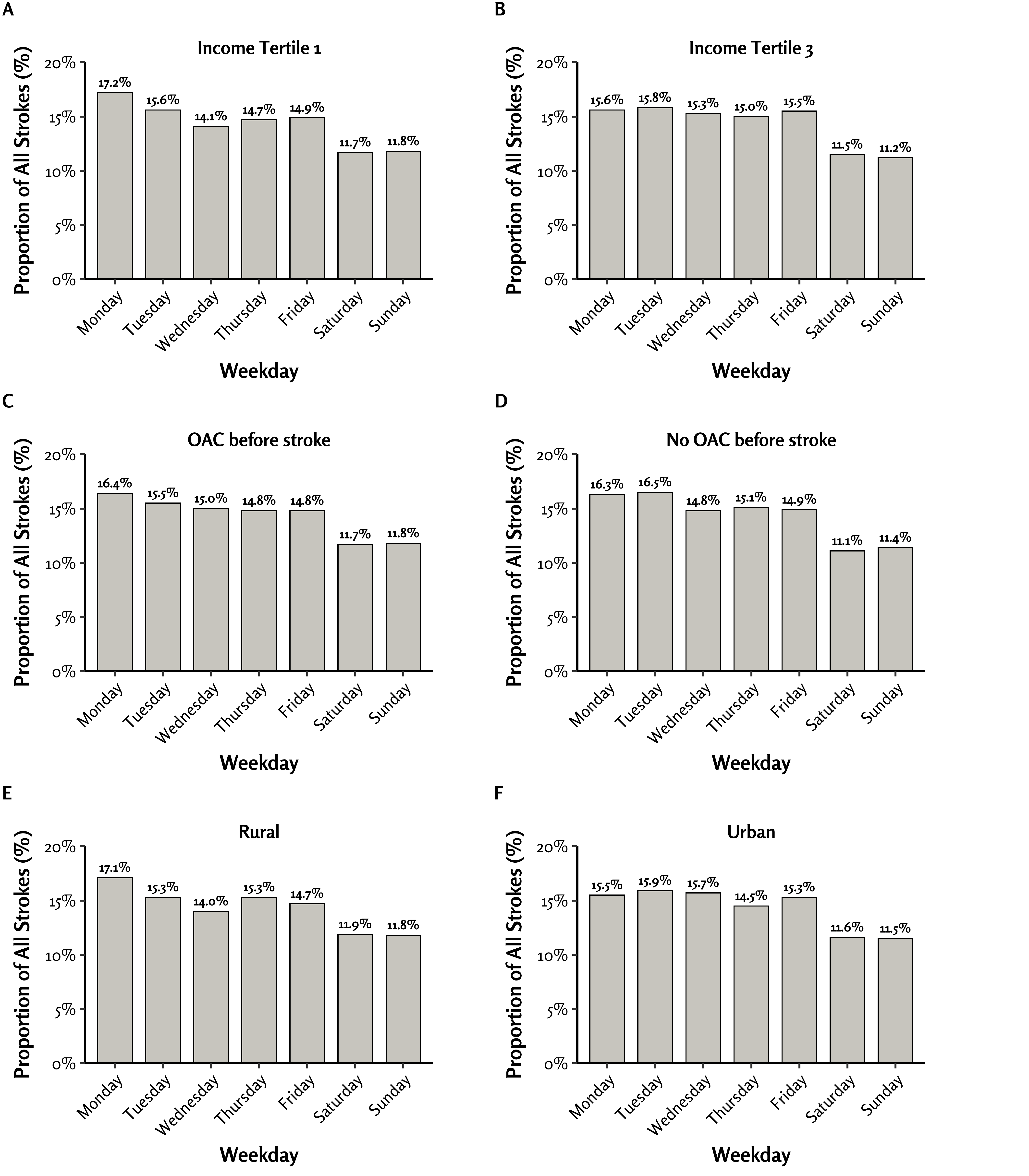

Supplement: Supplementary_Material.docx [file IANN_A_2667620_SM5922.docx]
